# Retraction Note to: MicroRNA-488 inhibits tongue squamous carcinoma cell invasion and EMT by directly targeting ATF3

**DOI:** 10.1186/s11658-022-00414-9

**Published:** 2022-12-27

**Authors:** Bingxia Shi, Wei Yan, Guolin Liu, Yanjun Guo

**Affiliations:** Oral and Maxillofacial Surgery, Cangzhou Central Hospital, No. 16 Xinhua West Road, Cangzhou, Hebei 061000 People’s Republic of China

**Retraction to:  Cellular & Molecular Biology Letters (2018) 23:28** **https://doi.org/10.1186/s11658-018-0094-0**

The Editor-in-Chief has retracted this article because of significant concerns regarding Figs. 1A, 1C, 2A, 2C, 3C, 4D and 5B, presented in this work, which question the integrity of the data. None of the authors have responded to any correspondence from the editor/publisher about this retraction.

